# Group 2i Isochrysidales thrive in marine and lacustrine systems with ice cover

**DOI:** 10.1038/s41598-024-62162-4

**Published:** 2024-05-20

**Authors:** Karen J. Wang, Yongsong Huang, Tyler Kartzinel, Markus Majaneva, Nora Richter, Sian Liao, Camilla S. Andresen, Flor Vermassen

**Affiliations:** 1 Department of Earth, Environmental and Planetary Sciences, Brown University, Providence, RI 02912 USA; 2 Institute at Brown for Environment and Society, Brown University, Providence, RI 02912 USA; 3 Department of Ecology, Evolution, and Organismal Biology, Brown University, Providence, RI 02912 USA; 4 Norwegian Institute for Nature Research (NINA), NO-7485 Trondheim, Norway; 5 Department of Marine Microbiology & Biogeochemistry, NIOZ Royal Netherlands Institute for Sea Research, 1790 AB Den Burg, The Netherlands; 6 Department of Chemistry, Brown University, Providence, RI 02912 USA; 7 Department of Glaciology and Climate, Geological Survey of Denmark and Greenland, Øster Voldgade 10, 1350 Copenhagen K, Denmark; 8 Department of Geological Sciences, Stockholm University, 106 91, Stockholm, Sweden; 9 Bolin Centre for Climate Research, Stockholm University, 106 91, Stockholm, Sweden

**Keywords:** Paleoclimate, Sea ice, Biomarker proxy, Alkenones, Isochrysidales, DNA, Biogeochemistry, Biogeochemistry, Cryospheric science, Palaeoceanography, Palaeoclimate, Microbial ecology

## Abstract

Global warming is causing rapid changes to the cryosphere. Predicting the future trajectory of the cryosphere requires quantitative reconstruction of its past variations. A recently identified sea-ice-associated haptophyte, known as Group 2i Isochrysidales, has given rise to a new sea-ice proxy with its characteristic alkenone distributions. However, apart from the occurrence of Group 2i Isochrysidales in regions with sea ice, and the empirical relationship between C_37:4_ alkenone abundance and sea-ice concentration, little is known about the ecology of these haptophyte species. Here, we systematically mapped the spatial and temporal occurrence of known Group 2i Isochrysidales based on environmental DNA in both marine and lacustrine environments. Our results indicate Group 2i is widely distributed in icy marine and lacustrine environments in both Northern and Southern Hemisphere, but is absent in warm environments. Temporally, Group 2i is part of the sea-ice algae bloom during the cold seasons, in contrast to other Isochrysidales that bloom in open waters during warm seasons. Our results indicate that ice is a prerequisite for the occurrence of the psychrophilic Group 2i haptophytes in marine and lacustrine ecosystems and further affirms its value for past ice reconstructions.

## Introduction

The cryosphere, including sea ice, lake ice, and glaciers, is a crucial element of the Earth's climate system that regulates the Earth’s heat budget. Pervasive changes in the cryosphere have already occurred under a warming climate^1^. Current Arctic sea ice cover has decreased to its lowest level since at least 1850 CE, with a continuous decrease in area and thickness in both summer and winter since 1978 when satellite observation began^1^. Loss of lake ice has also been observed over the recent decades, and the decrease in ice duration and thickness is projected to intensify at an unprecedented pace^2^. Accurate reconstruction of past cryosphere changes beyond instrumental records, especially during warmer periods in the geological past, is essential for calibrating future projections using climate models.

A new sea-ice proxy based on characteristic alkenone distributions produced by Group 2i Isochrysidales (an order of haptophytes) was recently proposed by Wang et al.^3^. Phylogenetically based on 18S rRNA gene, Isochrysidales have been classified into 3 groups, with Group 1 inhabiting freshwater and oligohaline environments, Group 2 in saline lakes and estuaries, and Group 3 in open ocean settings^4,5^. Group 2 can be further separated into ice-associated Group 2i and warm-season blooming Group 2w (e.g., *Isochrysis galbana*, *Ruttnera lamellosa*)^3–9^. Alkenones produced by Group 3 Isochrysidales have been widely used for paleo sea surface temperature (SST) reconstructions since the 1980s^10,11^. However, alkenones from sea-ice-laden oceans often lead to abnormal SST reconstructions with high values of %C_37:4_ (C_37:4_/( C_37:2_ + C_37:3_ + C_37:4_)) compared to mid-to-low latitude oceans, which was previously attributed to meltwater input and decreased salinity^12,13^. Recent culture experiments show that %C_37:4_ of alkenones produced by Group 2 and 3 Isochrysidales is not affected by salinity^14^. The unusually high %C_37:4_ observed in high-latitude oceans can be attributed to alkenone production by Group 2i during thawing of sea-ice which generally coincide with low surface salinity^3^. Cells and DNA sequences of Group 2i has been widely observed within sea ice^3^, and the correlation between occurrence of Group 2i and high %C_37:4_ has been used to reconstruct past changes in sea ice in regions such as the Gulf of Alaska and the Fram Strait^3,15, 16^. However, we have a limited understanding of the global distribution and ecology of Group 2i Isochrysidales in the natural environments. For instance, it was unclear whether Group 2i is present in the Antarctic region, and whether low temperature and ice are prerequisites for the presence of Group 2i in the marine and lacustrine environments. Further, there were no constraints on the growth conditions (e.g., temperature or salinity) or seasonality of Group 2i phytoplankton blooms in natural waters.

Here, we made the first global map of known occurrences of Group 2i and its habitable temperature ranges based on environmental DNA data sequenced from sediment samples collected in Baltic Sea, Chesapeake Bay, and Greenland fjords and re-analyzed environmental DNA data from published studies in global marine and lacustrine environments (Supplementary Data). We also examined the seasonal distribution of Group 2i during annual cycles to understand its role in bloom successions. We propose potential growth strategies adopted by Group 2i that enable its success in cold settings and discuss the implications for paleoclimate reconstructions.

## Results

### Spatial distribution of Group 2i Isochrysidales in marine and lacustrine environments

**Figure 1:**
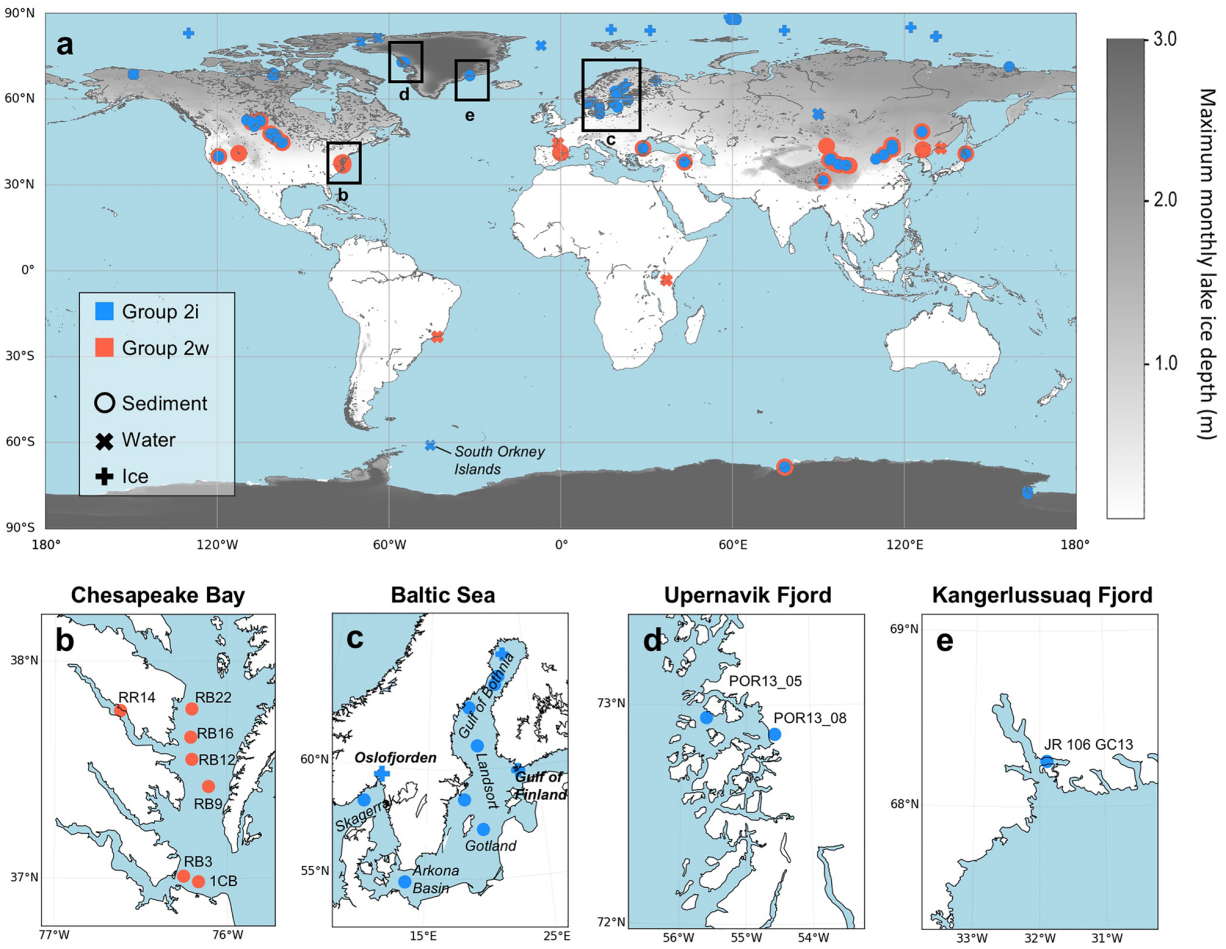
Global distribution of known Group 2w and 2i Isochrysidales based on environmental DNA from water, ice, surface and downcore sediment samples. (**a**) The contour shows maximum monthly lake ice thickness in each region averaged over 2005–2015, modeled in ERA5-Land^18^. (**b**-**e**) Zoomed in map of Chesapeake Bay, Baltic Sea, Upernavik Fjord and Kangerlussuaq Fjord sediment sample sites examined in this study. Samples illustrated in this figure are listed in Supplementary Data. The maps are generated using the Matplotlib Basemap Toolkit 1.4.1 (https://matplotlib.org/basemap/)^19^.

**Figure 2:**
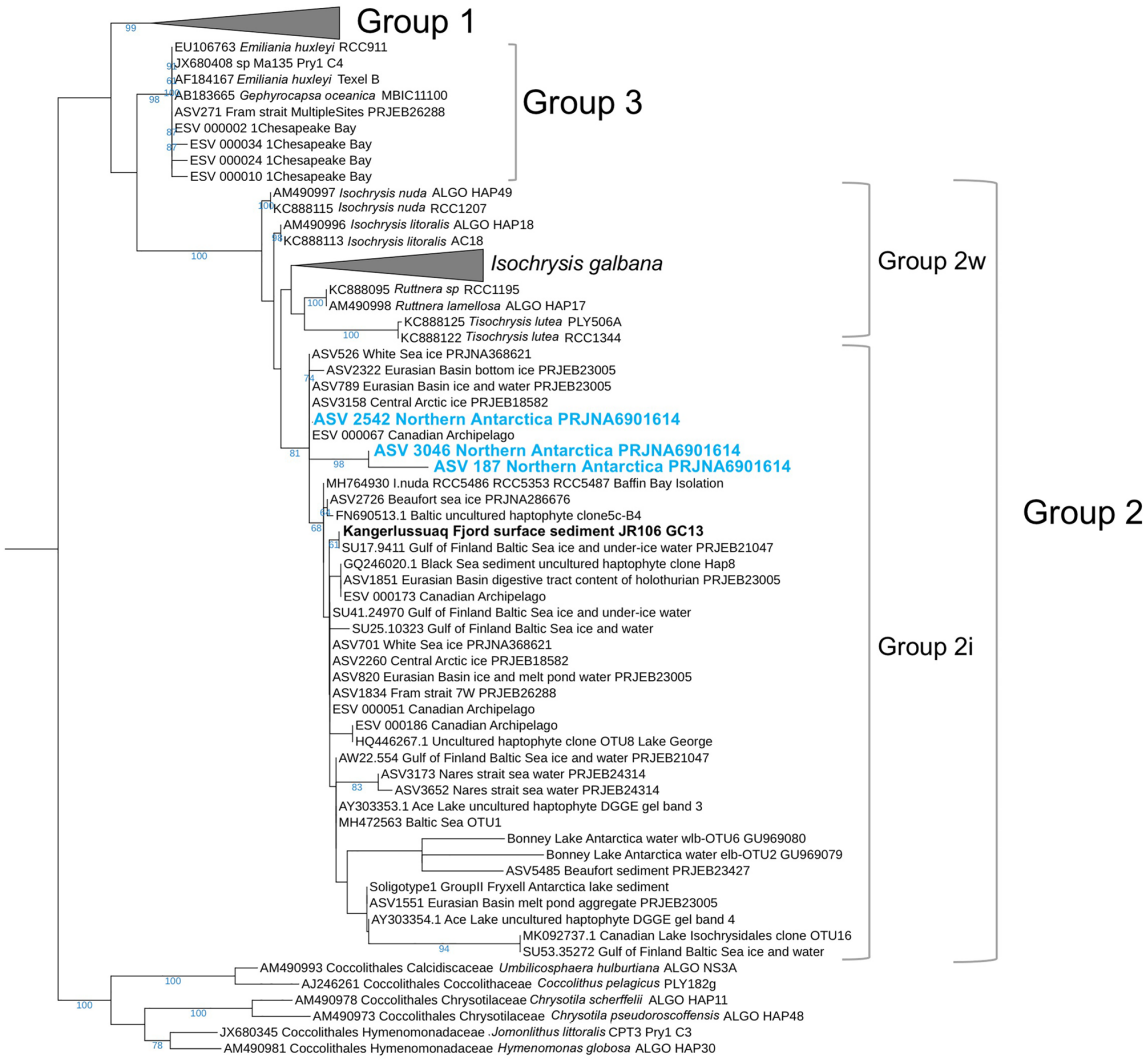
Phylogenetic tree of Isochrysidales 18S rRNA detected in the northern Antarctic Peninsula region. Bootstrap values that are > 50% are shown. Liu et al.^17^ collected surface water samples and water column samples in the northern Antarctic Peninsula region. Only three samples collected from water column at depth of 150 m, 200 m, 340 m at 60.85 S, 45.64 W recovered Isochrysidales DNA, all three ASVs falls into Group 2i.

We compiled global environmental DNA data from ice, water, and sediment samples in lacustrine and marine environments that targeted haptophytes, and mapped the global distribution of Group 2 Isochrysidales detected in different ocean environments (Fig. 1). Based on previous study, Group 2i is widely identified in seawater and ice samples in the Arctic Ocean, where other Isochrysidales are rare^3^. Here, we investigated DNA recovered from seawater samples collected in the Antarctic region and report the first identification of Group 2i in the Southern Ocean, from seawater samples collected during the austral summer in the northern Antarctic Peninsula region^17^ (Fig. 2). Group 2i DNA is found in water column samples from ocean adjacent to the South Orkney Islands. The water temperature at the time of sampling (January, 2018) range from − 0.5 to 3 °C and salinity range from 33.1 to 34.4 psu. No other Group 2 DNA was identified in the Southern Ocean water samples from studies we examined (Supplementary Data).

We analyzed Isochrysidales DNA and alkenone profiles from sediment samples in estuaries that encompass three temperature regimes: Greenland fjords, the Baltic Sea, and Chesapeake Bay. The Upernavik Fjord and Kangerlussuaq Fjord are located on the west and east coasts of Greenland, respectively (Fig. 1). The modern annual SST of the two sample sites ranges between − 2 to 4 °C (Fig. S1). Both fjords are major outlets of the retreating Greenland Ice Sheet^20,21^. Isochrysidales 28S rRNA gene sequences were recovered from downcore sediment samples from two core sites along Upernavik Fjord. Phylogenetic analyses based on 28S rRNA gene sequences show the Upernavik Fjord Isochrysidales sequences belong to Group 2i, as they clustered with Group 2i sequences recovered from surface sediment from Lake Fryxell^3,5^ (Fig. S2). Group 2i DNA was also detected through the 18S rRNA gene from surface sediment at the Kangerlussuaq Fjord (Fig. 2). The DNA of other Isochrysidales is not detected in Greenland fjords during this study. However, the alkenone profile from Kangerlussuaq Fjord suggests *Emiliania huxleyi* also exists at the site, and the alkenones are produced by both Group 2i and *Emiliania huxleyi*, as %C_37:4_ (14% in Kangerlussuaq sample) is not as high as in typical Group 2i cultures (~ 80% for strain RCC5486 under 3 °C^14^) and C_38_Me alkenones are relatively high as well (Fig. S3).

The Baltic Sea is an intra-continental shelf sea characterized by strong salinity and temperature gradients (Fig. S1). The south of the Baltic Sea is connected with the North Sea, where salinity is ~ 35 psu, in contrast to the northern end of Bothnian Bay and the Gulf of Finland where salinity is ~ 2 psu. The extent of sea ice from December to May in the Baltic Sea has large interannual variability and is an indicator of winter severity. During mild winters, only the Bothnian Bay and Gulf of Finland are ice-covered; during severe winters, ice covers the entire northern Baltic Sea extending to the south of Gotland and coastal areas of southern Baltic Sea^22,23^. All three groups of Isochrysidales were detected in the Baltic Sea region^24–26^. Skagerrak is dominated by alkenones produced by *Emiliania huxleyi* with small contributions from other Groups. From Skagerrak toward the Gulf of Bothnian estuary, the alkenone-producing community changed progressively (Fig. S3). The alkenone profile in the northern part of the Baltic Sea, where sea ice is more prominent, is dominated by Group 2i, in addition to Group 1 Isochrysidales in very low salinity (< 6 psu) waters^25,26^.

Chesapeake Bay’s annual SST ranges from 4 to 27 °C, and the seawater remains unfrozen during winter (Fig. S1). Isochrysidales 18S rRNA gene sequences were recovered from seven surface sediment samples in the lower part of Chesapeake Bay through next-generation sequencing (NGS). *Emiliania huxleyi* was detected in one sample near the mouth of the bay. The dominant amplicon sequence variants (ASVs) in all samples were *Isochrysis galbana* (a Group 2w Isochrysidales), which agrees with the microscopic identification conducted in a previous study^27^. The unidentified ASVs recovered all fall into Group 2w and have 99.2–99.7% similarity to *Isochrysis galbana* (Fig. S4). Importantly, no Group 2i was identified in these samples despite the recovery of abundant Group 2w and Group 3 Isochrysidales reads. The alkenone profile from the surface sediment is similar to the *Isochrysis galbana* culture with a small input from *Emiliania huxleyi* (Fig. S3).

**Figure 3:**
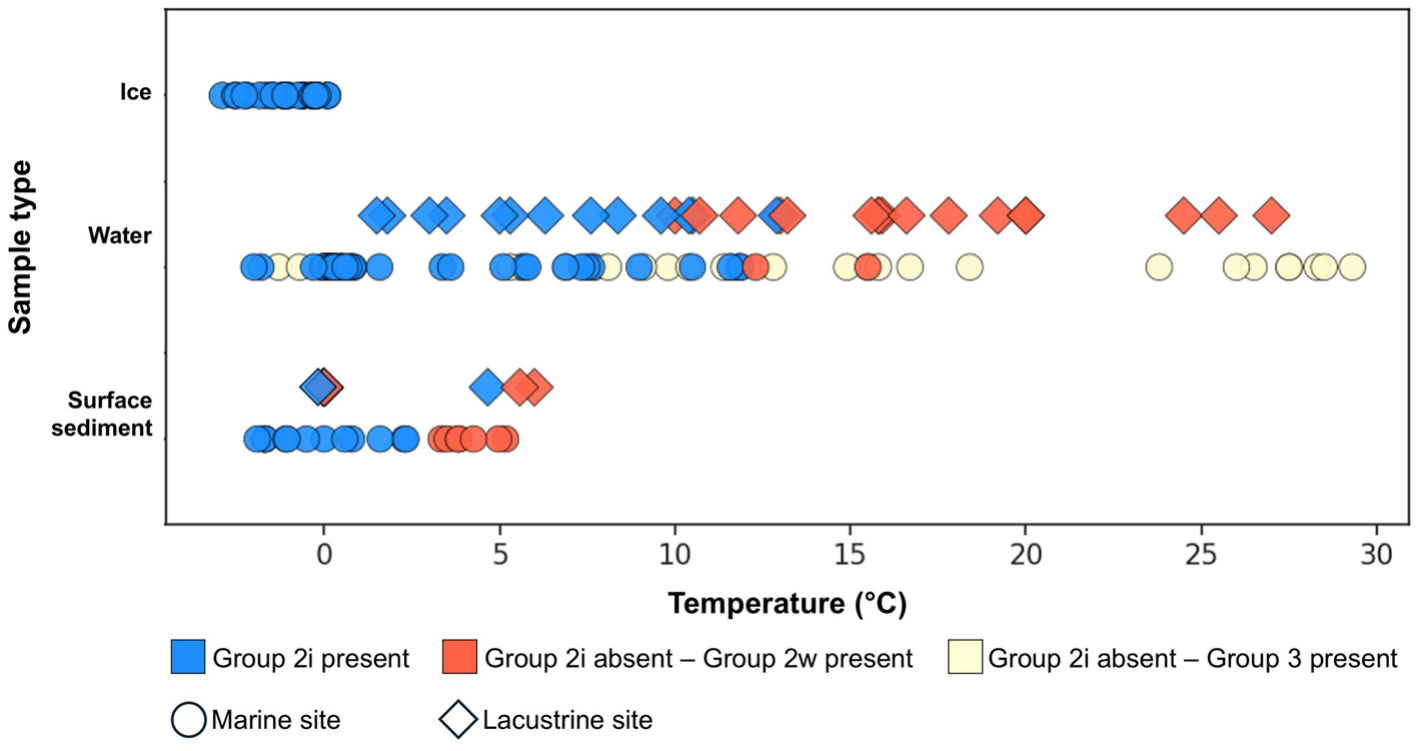
Temperature range of three groups of Isochrysidales. Scatter plot of temperature of ice, water, and surface sediment samples where Isochrysidales were detected in environmental DNA. Blue markers represent detection of Group 2i, red and yellow markers represent absence of Group 2i where there were positive detections of Group 2w and 3, respectively. Temperatures of ice and water samples are in situ temperature measured during sample collection. Temperatures of sediment samples are surface water temperature during coldest month calculated from ERA5 monthly averaged data^18^ over 2005–2015. Samples illustrated in this figure are listed in Supplementary Data.

Our results from various marine sites show that although Group 2i can occur across a wide salinity range from the typical open ocean (~ 35 psu) to oligohaline waters, as shown in the Baltic Sea and offshore Antarctic Peninsula, they are strongly associated with low temperature icy environments (Fig. 3). The absence of Group 2i in Chesapeake Bay demonstrates that they are limited to regions within or proximate to seasonal or perennial ice cover.

Group 2i is also widespread in icy lacustrine environments (Fig. 1). However, it is generally absent in freshwater lakes where Group 1 Isochrysidales flourish^5,28–30^, regardless of the temperature. Group 2i was only found in lacustrine environments where surface water temperatures fall to the freezing point during winter, except for one sample from Pyramid Lake, USA^4^ (Fig. 3). In modern samples from lakes with perennial ice cover (e.g., Lakes Fryxell and Bonney in the Antarctic Dry Valley), Group 2i was the only Isochrysidales found^5,31, 32^. In contrast, only Group 2w was found in temperate saline lakes in Spain, France, Tanzania, and Brazil, where lake water remains unfrozen throughout the year^33–35^.

### Phenology of Group 2i Isochrysidales

Within the same location where annual temperature varies from around freezing to over 10 °C, Group 2i only occurs in winter and spring when the water temperature is below ~ 12 °C (Fig. 3). Group 2i was detected in water samples with (1) temperatures below 0 °C, (2) temperature above 0 °C collected under-ice, or samples collected shortly after ice melt in spring; but 2i is absent in samples collected during the summer. Group 2i was not detected in any samples collected during an extensive survey of haptophytes conducted in the central North Pacific from 0° to 60° N along the transect of 170° W during the summer of 2014 where seawater temperature ranged between 9.8 and 29.3 °C^36^.

**Figure 4:**
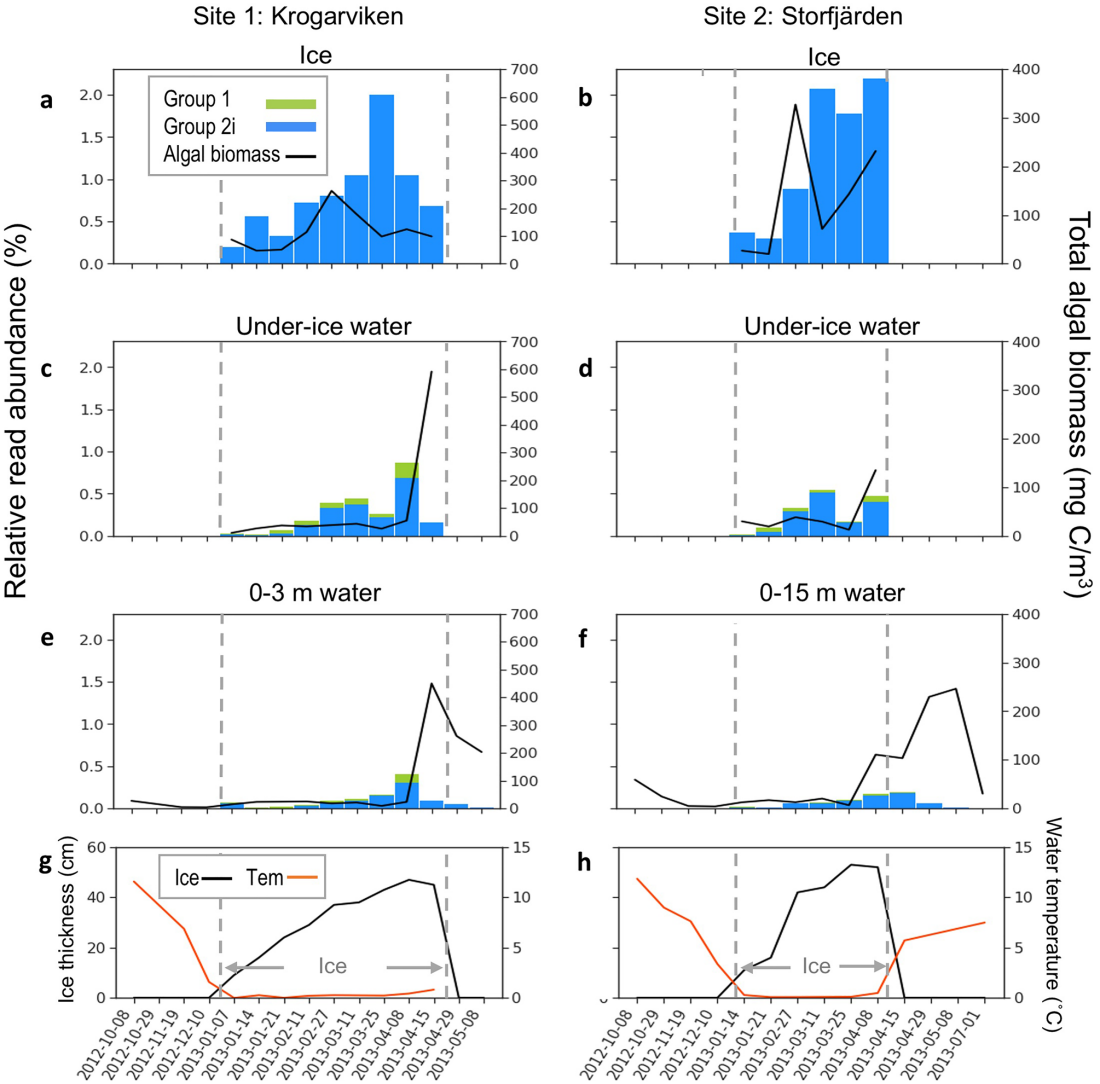
Isochrysidales detected from ice and water samples collected during Oct. 2012-Jul. 2013 at two sites from the Gulf of Finland. (**a**-**f**) The bar plots show Group 1 and 2i Isochrysidales relative read abundance to total Eukaryotes. The black lines show in situ total algal biomass measured during the sample collection. (**g**-**h**) Ice depth and under-ice water temperature measured at sample collection. The gray dashed lines indicate the time window during which ice existed. Raw NGS data from: Enberg et al.^23^.

We examined Isochrysidales DNA data from ice and seawater samples collected across seasonal cycles from two regions in the Baltic Sea, Gulf of Finland and Oslofjorden. Isochrysidales sequences were found in the ice and water column during the cold seasons (Oct.-May) in the two monitored sites at the northwest of the Gulf of Finland—Krogarviken and Storfjärden by Enberg et al.^25^. Both of the sites were covered by sea ice from January to April, and the total algal biomass was higher in the ice than in the water column during the ice-covered season and slowly increased as ice thickness increased^25^ (Fig. 4). Isochrysidales communities in the ice, under ice water (collected at the ice-water interface), and water column were composed of Group 2i and Group 1 Isochrysidales. Group 2i was the predominant haptophyte in the ice samples based on relative read abundance, where the brine salinity ranged widely from approximately 4–45 psu. It made up 40–85% of the total haptophyte read in Krogarviken and 58–86% in Storfjärden, and accounted for up to 0.2–2.0%, and 0.3–2.2% of the total Eukaryotic reads in the Krogarviken and Storfjärden sea ice from January to April (Fig. 4). In contrast, zero or only a few (< 5 reads) Group 1 DNA sequences were detected in each ice sample, the majority of Group 1 was detected in very low salinity (~ 4 psu) water samples. Group 2i’s relative read abundance to total eukaryotes and absolute read count were lower in the water column than in the ice from February through early April, and the abundance in the water decreased sharply as the ice started to melt in mid-April. The Group 2i population in the water column decreased as water temperatures rose and was absent in most of the water samples collected during July-November at both sites (Fig. 4).

**Figure 5:**
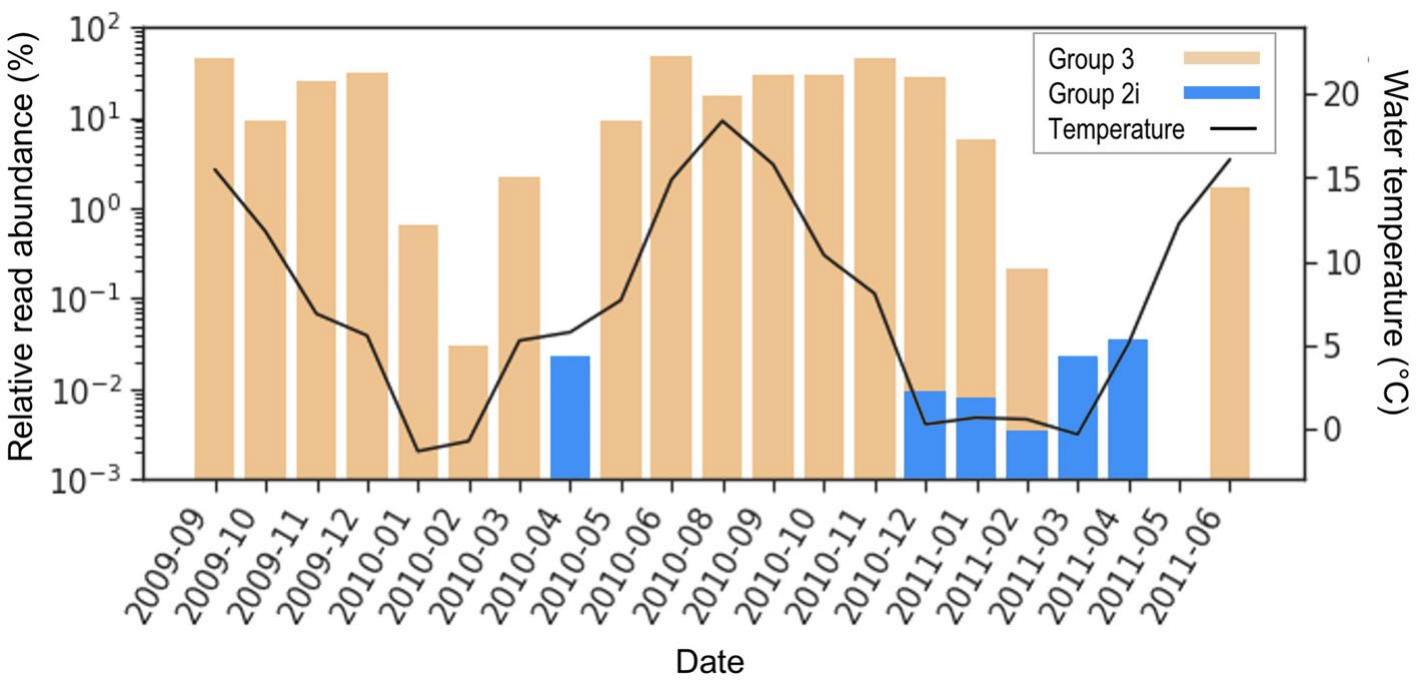
Isochrysidales successions in Oslofjorden, Norway during Sept. 2009-June 2011. Barplot of Group 2i and *Emiliania huxleyi* relative read abundance to total haptophytes from ice and water samples collected, note the y-axis is in log-scale, and the bars representing the two lineages are overlaid instead of stacked. The black line represents in situ water temperatures at 1 m depth when samples were collected. Raw NGS data from: Egge et al.^22^.

Egge et al.^24^ collected water samples in outer Oslofjorden (59.19° N, 10.69° E) at 1 m depth monthly and sequenced haptophytes through NGS from September 2009 to June 2011. Group 1, 2i, 2w, and 3 were all detected at this site. *Emiliania huxleyi* was detected in all months except for April and represented one of the predominant haptophyte species during the high-temperature months from summer to early autumn (Fig. 5). Group 2w was detected in September 2009 and May 2011 when the surface temperature reached 16 °C. Group 2i, in contrast, was absent during the warm season and only detected during December 2010-April 2011. Maximum ice cover during the 2009/2010 winter season occurred in February 2010, with ice cover extending to Kattegat and Skagerrak and extensive sea ice formed in Upper Oslofjorden^37^. The relatively low sequence reads of Group 2i detected in April 2010 at the sampling site suggest that Group 2i might have been transported from Upper Oslofjorden upon ice melt. Winter 2010/2011 experienced a severe ice season with the largest maximum ice extent since the 1990s^22,23^.Coastal ice formed in the southern Baltic Sea under a prolonged low temperature period^22,23^. Group 2i was detected in all samples collected during December 2010 -May 2011. The sequences in Oslofjorden and Gulf of Finland further demonstrate the substantial differences in the key environmental controls on the bloom phenology of the three groups.

## Discussions

### Growth strategies

The global distribution of Group 2i Isochrysidales shows that the lineage is restricted to marine and lacustrine habitats with seasonal or perennial ice cover. The time series of water and ice samples from the Baltic Sea shows that Group 2i is a part of the ice algae community and blooms before other Isochrysidales in the early spring. Potential growth strategies that contribute to its prosperity under cold climates in both hemispheres may include 1) formation of resting stages; 2) mixotrophy; 3) a preference for low growing temperatures and adaptability to a wide range of salinities.

Forming of cysts was observed in Group 2 species, which allows the cells to stay in sea ice or surface sediments for a long time^38^. In an enrichment experiment, Group 2i was triggered out of its resting stage (during which the cells were not active and alkenone production was paused) in the sediment by increasing the light intensity from 100 to 200 μmol m^2^s^−1^^39,40^. Group 2i likely forms cysts in the sediment and sea ice during prolonged winters when light availability is limited, and can be activated when light availability increases. This may have occurred in the Canadian Arctic Archipelago, Baffin Bay, and Greenland fjords, where the bathymetry of these regions enhances vertical mixing from tidal energy which can re-suspend cysts from the sediment and activate them^3,41, 42^.

Group 2i could potentially use mixotrophy to compensate for reduced photosynthesis under light-limited conditions. Multiple Isochrysidales species are thought to be capable of mixotrophy, including *Emiliania huxleyi*, *Gephyrocapsa oceanica*, *Isochrysis galbana*, and *Tisochrysis lutea*^43–46^. Increased biomass production and nutrient conversion efficiency under mixotrophic conditions has been reported in Group 2 species *Isochrysis galbana* and *Tisochrysis lutea*^44,45^. Mixotrophy of Group 2i has been observed in the McMurdo Dry Valley Lake located in Antarctica^32^. Culture enrichment of Isochrysidales isolated from this site could grow in either dark or light conditions but achieved optimal growth under mixotrophic conditions with organic carbon sources such as cereal grass, rice, and yeast extract^32^. Group 2i Isochrysidales take up a significant proportion (80% based on DNA sequencing) of the mixotrophic community in the McMurdo Dry Valley Lake and is an important primary producer in this lake. Moreover, the gene copy abundance of Group 2i measured through qPCR is not correlated with light availability in this lake^32,47^. The capability of mixotrophy could give Group 2i an advantage during the early stages of the phytoplankton bloom succession around ice melt where light availability was still low. Mixotrophy could potentially compensate for photosynthesis for Group 2i when photosynthetically active radiation was relatively low^31,32^.

Culture experiments also demonstrate Group 2i Isochrysidales’ preference for low growing temperatures and adaptability to a wide range of salinities. Liao and Huang^14^ cultured the Group 2i strain RCC5486 (isolated from sea ice samples from Baffin Bay^42^) at different temperatures from 0 to 12 °C at 31 ppt under light: dark cycle of 16:8 h and found that RCC5486 growth rates were high at 0, 3, 6, 9 °C, but failed to grow at 10.5 and 12 °C. Liao and Huang^14^ also tested different salinities (15, 21, 26, 31, and 38 ppt) under 3 °C and did not observe a significant difference in growth rate at different salinities. The preference of low temperature and flexibility with salinity enable Group 2i to survive in sea ice brine channels, melt ponds, and under-ice water, where the temperature is low and salinity fluctuates greatly when ice forms and melts. The temperature preference explains why Group 2i is not detected in sediments from Chesapeake Bay and temperate lakes and water columns during summer, i.e. Lake George and the Baltic Sea^40^.

### Habitats, bloom successions and implications for paleoclimatology

The seasonal succession of Group 2i in the Gulf of Finland suggests that it does not solely live in the meltwater, in the under-ice water, or was merely “trapped into” the sea ice. Instead, Group 2i was concentrated into the sea ice during ice formation via certain selective mechanisms, and can thrive in the brine channels under fluctuating salinity^25^. In contrast to *Emiliania huxleyi*, which blooms during warmer temperatures, Group 2i was absent in water samples collected during the summer months in the Gulf of Finland and outer Oslofjorden. Group 2i is not a part of the summer phytoplankton bloom; instead, they are part of the ice algae assemblage and potentially seeded the under-ice bloom through brine drainage in the spring. Group 2i likely permeates all three phases of the ice algal bloom from pre-bloom, bloom, to post-bloom. It is likely most prominent in the first two phases before the ice starts to break up based on two observations: 1) Group 2i was the most abundant lineage among haptophytes in the sea ice during the entire ice season in Gulf of Finland; and 2) active Group 2i cells were isolated from an ice core in Baffin bay during pre-bloom and bloom development^25,42^. The exact timing of Group 2i growth and its role in the bloom succession likely differs depending on available sunlight, ice properties, temperature, and nutrient conditions.

Similar Isochrysidales bloom successions exist in lacustrine environments with seasonal ice cover. In a water column time series from Lake George during the spring, two non-overlapping Isochrysidales blooms were observed^40^. The early bloom that consisted of Group 2i peaked shortly after ice melt when the surface water was around 7 °C, but the population quickly declined as water temperature continued to rise^40^. In contrast, the later bloom consisted of Group 2w, and its DNA read abundance peaked around 15 °C. The two separate blooms are also evident in different alkenone profiles, which are dominated by C_37:4_ and C_37:3,_ respectively ^39^.

A holistic picture of Group 2i's ecology greatly extends alkenones and environmental DNA as paleoclimate proxies: (1) It reaffirms using the presence of Group 2i and elevated %C_37:4_ as sea-ice proxy in high latitude oceans for both Northern Hemisphere and Southern Hemisphere; (2) In temperate regions with seasonal ice where both 2i and 2w are present in saline lakes, interpretation of %C_37:4_ is less straight forward, as Group 2w is also capable of producing variable amounts of C_37:4_. However, C_39:4_Me alkenone has been found exclusively in 2i Isochrysidales and is not present in 2w species^9,14^. Thus, the relatively abundance of C_39:4_Me alkenone (i.e., %C_39:4_) may be better suited for reconstructing past lake ice coverages. (3) In settings where only Group 2i or 2w is present, alkenones may allow reconstruction of cold or warm season temperatures, respectively. For example, in warm settings with no seasonal ice cover, such as Spanish lakes and Chesapeake Bay, alkenones are produced by 2w species during the warm season. In such settings, alkenone unsaturation ratios should primarily reflect warm season temperatures.

## Conclusions

DNA data from global marine and lacustrine environments indicate that Group 2i Isochrysidales thrive in marine and lacustrine environments with seasonal or perennial ice cover across both hemispheres, but are absent from warm environments where the water body remains unfrozen throughout the year. Group 2i is well adapted to a wide range of salinity and may be capable of mixotrophy and forming resting cysts. Such unique traits may have enabled Group 2i to thrive in brine channels within sea ice. Group 2i is a part of the sea-ice algae community that blooms in late winter and/or early spring, which is different from Group 3 and Group 2w that bloom during warm seasons. Our results reaffirm the potential of using the characteristic alkenone distributions found in Group 2i for past ice reconstructions.

## Methods and materials

### Surface sediments from the Chesapeake Bay and Greenland fjords

Sediment samples from the Baltic Sea, Chesapeake Bay, USA, and two Greenland Fjords were collected for DNA and alkenone analyses (Table S1). Surface sediment samples were collected across the Baltic Sea from Skagerrak to the Bothnian Sea during expeditions M86/1 (R/V Meteor; November 2011), P435 (R/V Poseidon; June 2012), 6EZ1215 (R/V Elisabeth Mann Borgese; July 2012), EMB046 (R/V Elisabeth Mann Borgese; May 2013) through multi- corer^48^. Surface sediment samples were collected in the Chesapeake Bay from July–September 2017 and kept frozen prior to DNA extraction. Two sediment cores (POR13_05 and POR13_08) from Upernavik Fjord were collected in August 2013 onboard R/V Porsild with Rumohr corer^20^. One surface sediment sample from Kangerlussuaq Fjord was collected during JR106 (James Clark Ross; 2004).

### DNA sequencing

Surface sediment samples from the Baltic Sea are sequenced through Sanger with haptophyte-specific primers targeting 18S rRNA as described in Kaiser et al.^26^. In short, DNA was extracted and purified from 0.5 g of dried sediments. One round of PCR was performed with primer pair Prym-429F and Prym-887R^49^. The PCR products were run on gel electrophoresis, and gel bands with targeted length were purified and cloned. The clone products were sequenced on an ABI 3730XL capillary sequencer (Applied Biosystems, Foster City, CA). Operational Taxonomic Units (OTUs) were clustered by UCLUST. Representative sequences of each OTU were deposited in GenBank under accession numbers MH472563-MH472566.

The surface sediment samples from the Chesapeake Bay and Kangerlussuaq Fjord were sequenced through NGS with haptophyte-specific primers targeting 18S rRNA following methods described in Wang et al.^3^, and stored under accession number PRJNA835469 in GenBank. In short, DNA was extracted from the samples, followed by a two-step PCR. The first round of PCR used an adapted version of the primer pair 528Flong and PRYM01 + 7^50^ followed by a second round of PCR which gives each sample a unique index sequence. The cleaned and normalized amplicons were then sequenced on an Illumina Miseq with V2 600-Cycle Kit (San Diego, CA). Sequences were verified using FastQC v0.11.8 and clustered into Amplicon Sequence Variants (ASVs). Taxonomy of ASVs were assigned using the SILVA reference database.

Water and ice sample collection and sequencing in the Gulf of Finland is described in Enberg et al^25^. Relative read abundance (RRA) to total haptophyte of Eukaryotes for the *i*th OTU is defined as: ${RRA}_{i}=\frac{1}{S}\sum_{k=1}^{S}\frac{{n}_{i,k}}{\sum_{i=1}^{T}{n}_{i,k}}\times 100\%$, where S is the number of samples, *T* is the total number of haptophyte or Eukaryotes OTU, ${n}_{i,k}$ is the number of sequences of OTU *i* in sample *k*^51^. The presence/absence of Isochrysidales sequences was defined as if any ASV or OTU were identified as Isochrysidales in the sample.

The downcore sediment samples from Upernavik Fjord were sequenced on an Illumina MiSeq 500-cycle paired-end run with haptophyte-specific primers (HAP_LSU_F and LHapto20R_bis) targeting D1-D2 region of 28S rRNA following methods described in Richter et al.^5^. Isochrysidales sequences were selected through BLAST and aligned to the 28S rRNA reference sequences in Richter et al.^5^ by PyNast. Maximum-likelihood phylogenetic trees for 18S and 28S rRNA were built by RAxML under GTRCAT approximation with 1000 bootstrap on CIPRES to determine the sub-groups of the Isochrysidales sequences^52,53^. We also reanalyzed environmental sequences recovered in seawater samples from Southern Ocean near Antarctica Peninsula (Supplementary Data), following methods described in Wang et al.^3^. Sequences classified as Isochrysidales are included in phylogeny tree shown in Fig. 2.

### Alkenones analyses

Freeze-dried sediment samples were extracted by an accelerated solvent extraction system with DCM: methanol mixture (9:1, v/v). The total lipid extraction was further separated into three fractions of increasing polarity by silica gel columns eluting with hexane, DCM, and methanol subsequently. The mid-polar fractions containing alkenones were analyzed on a gas chromatography–flame ionization detection Agilent 7890N Series instrument with mid-polarity column RTX-200 (105 m × 250 μm × 0.25 μm) with the oven program described in Zheng et al.^54^.

### Supplementary Information

Supplementary Information 1.

Supplementary Information 2.

## Data Availability

Representative sequences from each OTU recovered from Baltic Sea sediment using Sanger sequencing are stored in NCBI GenBank under accession number MH472563-MH472566. Raw sequencing data from sediment samples in Chesapeake Bay and Kangerlussuaq Fjord are stored in Sequence Read Archive (SRA) database of NCBI under accession number PRJNA835469. List of published datasets re-investigated in this study are included in supplementary materials. Processed data outcomes are available upon request.
