# Supplementary material for: Group 2i Isochrysidales thrive in marine and lacustrine systems with ice cover
Source: Sci Rep. 2024 May 20;14:11449. doi: 10.1038/s41598-024-62162-4 (PMC11106077; doi:10.1038/s41598-024-62162-4)
Supplement: Supplementary file 2 — Supplementary Information 2. [file 41598_2024_62162_MOESM2_ESM.pdf]

Supplementary Material

Karen J Wang[1,2], Yongsong Huang[1,2], Tyler Kartzinel[2,3], Markus Majaneva[4], Nora Richter[1,2,5], Sian Liao[2,6], Camilla S Andresen[7], Flor Vermassen[7,8,9]

1. Department of Earth, Environmental and Planetary Sciences, Brown University, Providence, RI, 02912, USA
2. Institute at Brown for Environment and Society, Brown University, Providence, RI, 02912, USA
3. Department of Ecology, Evolution, and Organismal Biology, Brown University, Providence, RI, 02912, USA
4. Norwegian Institute for Nature Research (NINA), NO-7485, Trondheim, Norway
5. Department of Marine Microbiology & Biogeochemistry, NIOZ Royal Netherlands Institute for Sea Research, 1790 AB Den Burg, the Netherlands
6. Department of Chemistry, Brown University, Providence, RI, 02912, USA
7. Department of Glaciology and Climate, Geological Survey of Denmark and Greenland, Øster Voldgade 10, 1350, Copenhagen K, Denmark
8. Department of Geological Sciences, Stockholm University, 106 91, Stockholm, Sweden
9. Bolin Centre for Climate Research, Stockholm University, 106 91, Stockholm, Sweden

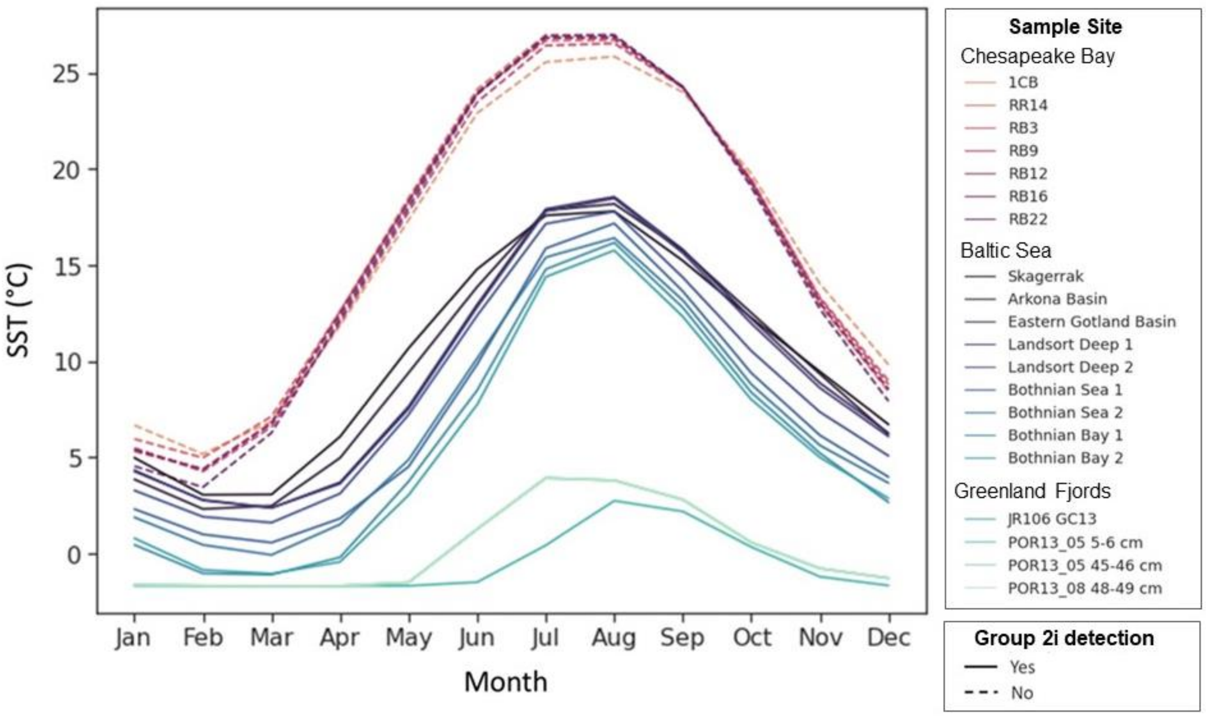

Figure S1. Monthly SST of sediment sample sites in Chesapeake Bay, Baltic Sea, and Greenland fjords. The climate data is from ERA5[1] monthly averaged data from 2005-2015.

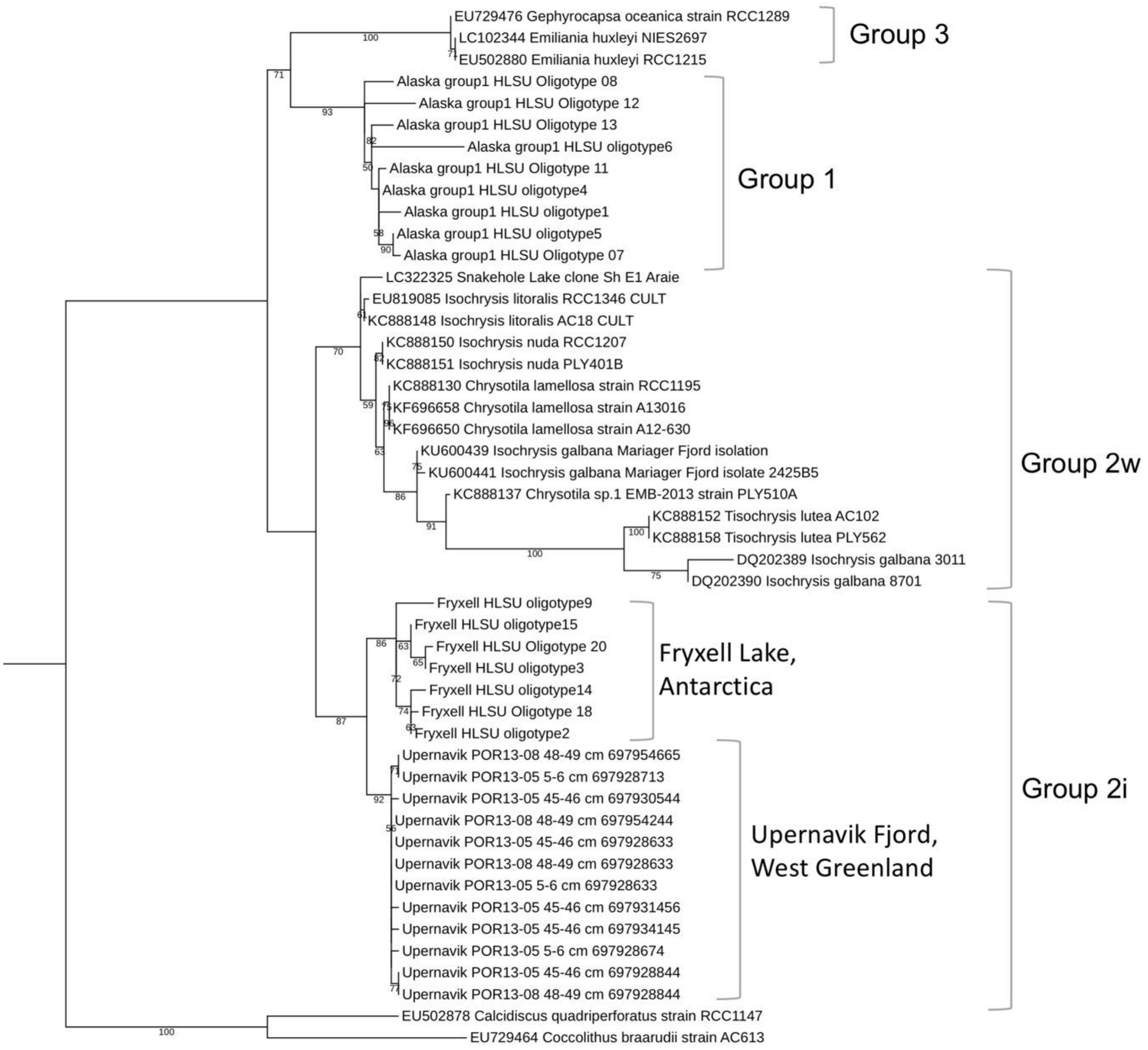

Figure S2. Phylogenetic tree of Isochrysidales 28S rRNA. Bootstrap values that are >50% are shown. Sequences found from Upernavik Fjord are clustered with Group 2i detected from Fryxell Lake.

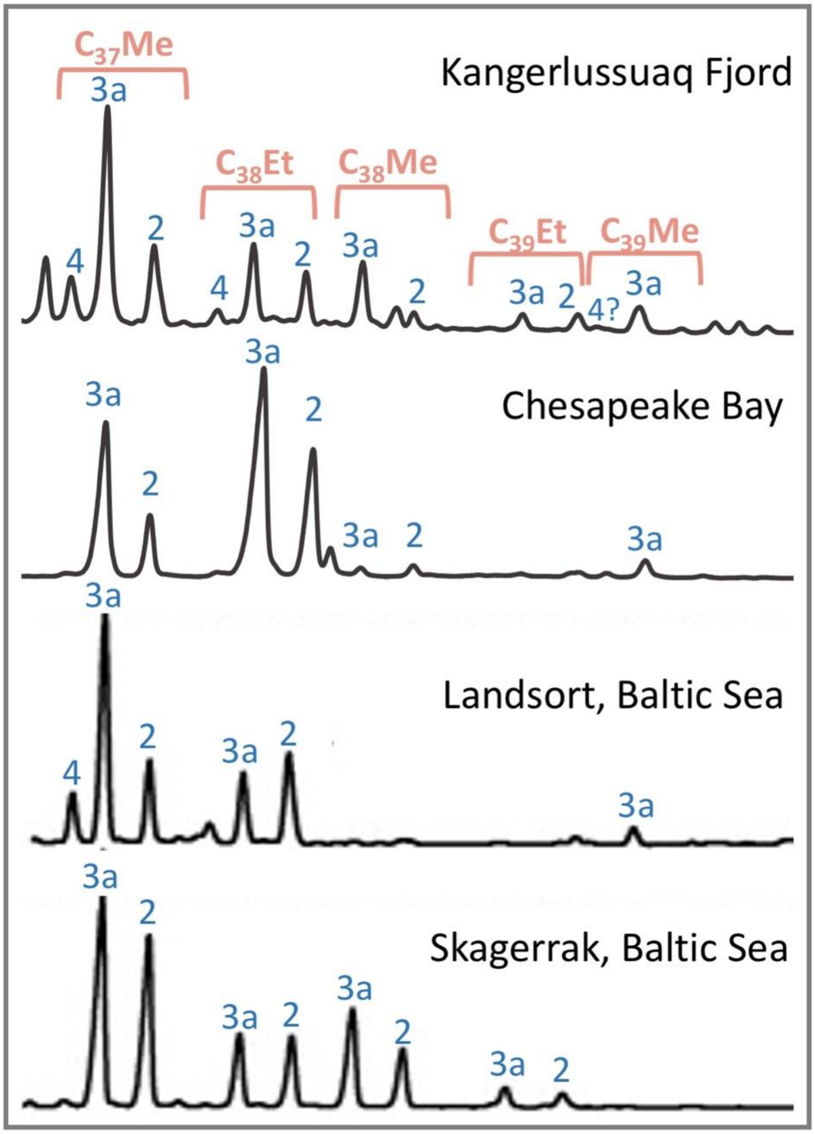

Figure S3. Representative GC-FID chromatograms of alkenones in surface sediment from Kangerlussuaq Fjord (GC13), Chesapeake Bay (RB12), central Baltic Sea (Landsort Deep), south Baltic Sea (Skagerrak)

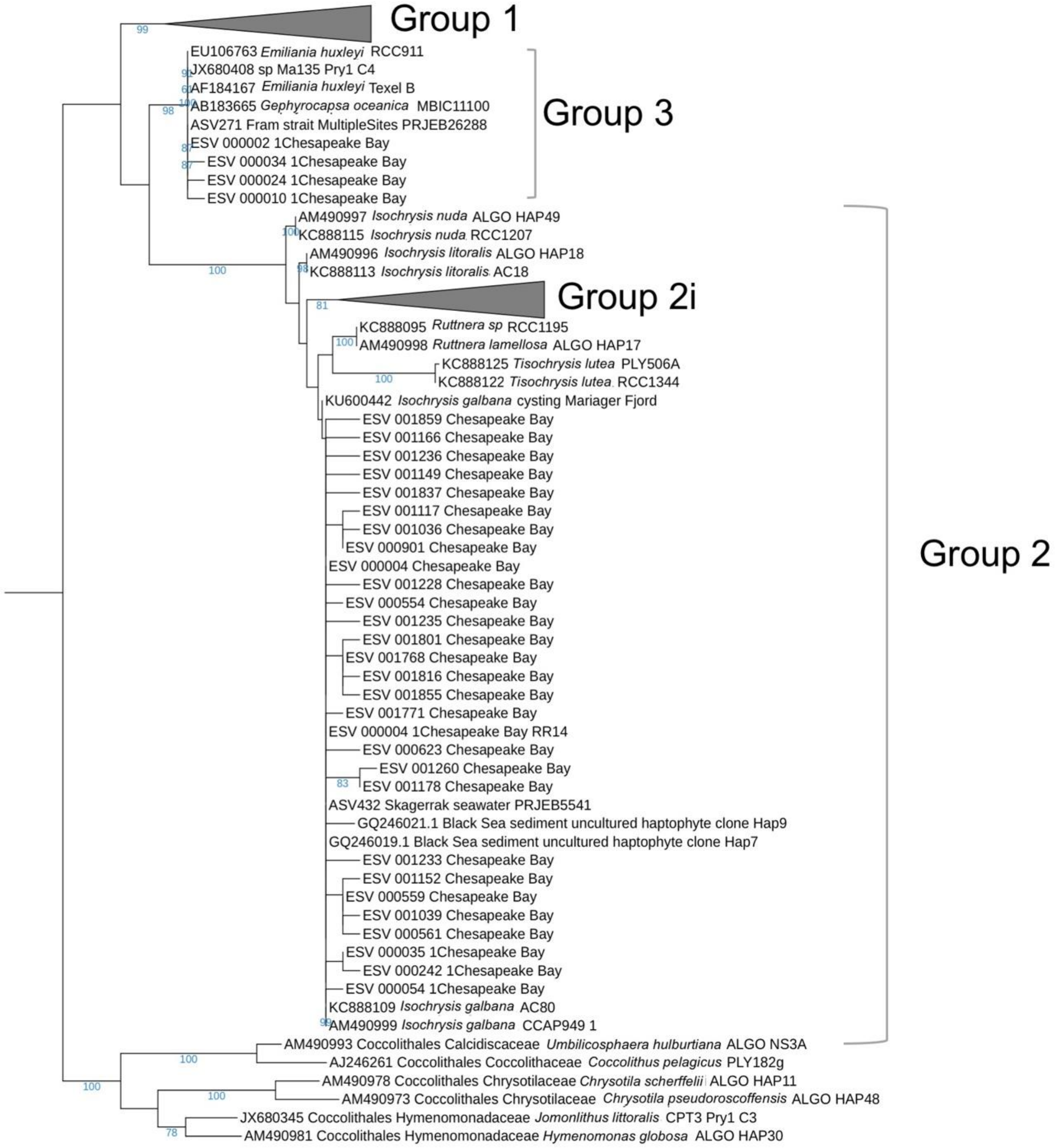

Figure S4. Phylogenetic tree of Isochrysidales 18S rRNA detected in Chesapeake Bay. View of the same phylogenetic tree with expanded Group 2i is shown in Fig. 1. Bootstrap values that are >50% are shown. Sequences from the Chesapeake Bay are clustered with cultured *Emiliania huxleyi* and *Isochrysis galbana*.

Table S1. Sediment samples examined in this study and the presence/absence of Isochrysidales (Y=yes, N=no).

Sample ID	Region	Lat.	Lon.	Depth	Sequencing method	Group 1	Group 2w	Group 2i	Group 3
1CB	Chesapeake Bay, USA	36.985	-76.166	Surface	528Flong and PRYM01+7; NGS	N	Y	N	Y
RR14		37.774	-76.619	Surface		N	Y	N	N
RB3		37.011	-76.252	Surface		N	Y	N	N
RB9		37.425	-76.109	Surface		N	Y	N	N
RB12		37.549	-76.205	Surface		N	Y	N	N
RB16		37.653	-76.209	Surface		N	Y	N	N
RB22		37.783	-76.203	Surface		N	Y	N	N
Skagerrak	Baltic Sea	58.429	9.477	Surface	Prym-429F and Prym-887R; Sanger	N	N	Y	N
Arkona Basin		54.852	13.433	Surface		Y	N	Y	N
Eastern Gotland Basin		57.083	19.985	Surface		Y	N	Y	N
Landsort Deep 1		57.267	19.796	Surface		Y	N	Y	N
Landsort Deep 2		58.641	18.265	Surface		Y	N	Y	N
Bothnian Sea 1		61.085	19.579	Surface		N	N	Y	N
Bothnian Sea 2		62.845	18.889	Surface		Y	N	Y	N
Bothnian Bay 1		63.833	21.583	Surface		Y	N	Y	N
Bothnian Bay 2		64.204	22.029	Surface		Y	N	Y	N
JR106 GC13	Kangerlussuaq Fjord	68.26	-31.89	Surface	528Flong and PRYM01+7; NGS	N	N	Y	N
POR13_05 5-6 cm	Upernavik Fjord	72.945	-55.62	Core, 5-6 cm	HAP LSU F, LHapto20R bis; NGS	N	N	Y	N
POR13_05 45-46 cm		72.945	-55.62	Core, 45-46 cm		N	N	Y	N
POR13_08 48-49 cm		72.869	-54.558	Core, 48-49 cm		N	N	Y	N

Reference

1. Hersbach, H. *et al.* The ERA5 global reanalysis. *Q.J.R. Meteorol. Soc.* **146**, 1999–2049 (2020).
